# Gun-Shot Wounds of the Small Intestines

**Published:** 1884-08

**Authors:** Charles T. Parkes

**Affiliations:** Professor of Anatomy in Rush Medical College, Chicago, Ill.


### SELECTIONS.

GUN-SHOT WOUNDS OF THE SMALL INTESTINES. *By* CHARLES T. PARKES, M. D., *Professor of Anatomy in Rush Medical College, Chicago, Ill.*

[*Address of the Chairman of the Section on Surgery and Anatomy.*]

[*Continued from July Number.*]

Eleventh. Is not the moral effect of the assurance to the patient, that he will be placed in a condition most likely to lead to his recovery, a good substitute for the mental depression accompanying the general and popular conviction that these wounds mean certain death?

---

EXPERIMENTS ON GUN-SHOT WOUNDS OF THE ABDOMINAL CAVITY.

[*Appended to Dr. Parkes' Address.*]

EXPERIMENT NO. 1.

Wednesday, November 14, 1883.—Long, lean and lank setter; about 30 lbs.; in rather poor condition. Etherized with common ether and shot through abdomen with a No. 32 cal. ball, which passed directly through anterior wall to the innominate bones. Upon opening abdomen found ileum perforated about the middle and at the ileo-cæcal valve, and slightly grazed at another point. Extravasation of intestinal contents at each perforation. Some entozoa. The two perforated regions were resected and continuity of ileum re-established by Lembert's intestinal stitch. The grazed portion was closed by continued suture. Intestines washed with a feeble solution of carbolic acid, and returned to abdomen; and the external wound closed by two sets of sutures, one set through muscular walls, including peritoneum, and the other uniting the skin.

Drainage tube inserted, and wound dressed antiseptically with gauze, cotton, etc.

Entire operation lasted about one hour, and conducted antiseptically, abdomen being shaved, washed and irrigated with carbolized solution, etc. The animal waked considerably shocked. Gave rectal injection of alcohol and water (1-2 1-2), about 3 drachms, and in half an hour gave 10 drops of laudanum. The dog seemed very bright. At 6 P. M. was considerably weakened; respiration very rapid, with much febrile excitement. About three-quarters of a pint of warm milk per stomach-tube, twelve drops of laudanum, and about a quarter-grain of morphia hypodermically. Tied him under register well bedded in a large comforter, and covered him with a coat.

Thursday, November 15.—Dog died between midnight and morning. Post-mortem. Abdomen filled with bloody serum and intestines inflamed and badly-smelling; kidneys a bright blue, and rectum filled with hardened fæces. Dog had vomited during the night a quantity of hair and some pieces of cartilage, etc. Died of shock. Wounds of intestine well agglutinated.

EXPERIMENT NO. 2.

Friday, November 16.—The ball (32) severed the aorta.

EXPERIMENT NO. 3.

Saturday, November 17.—(Ball 32 cal.) Dog etherized, shaved and shot very near abdominal margin. Upon opening abdomen (incision 3½ inch), but one loop of gut was found perforated. This was excised, and the continuity of intestine restored by eighteen individual silk stitches, which brought serous surface to serous surface, the greatest difficulty being encountered at the mesenteric attachment to the gut on account of the fat which lay between the two layers of peritonæum and adherent to the gut itself. Extravasation of intestinal contents. Tape worm.

Wound closed with heavy silk and dressed antiseptically. Gave 15 gtt. laudanum at night.

November 18.—Animal seems bright, but disposed to remain quiet, drinks plenty of water and urinates freely, Respiration hurried and febrile action high.

November 19.—Seemed very well, and partook of some milk during morning, but began vomiting in the P. M. a sour, watery and greenish fluid. Gave per rectum about ¼ grain morphiæ sulphas at night, and left him sleeping comfortably.

November 20.—Seemed quite lively and comfortable, the dressings having been removed the day before, and a large body bandage applied. About 11 A. M. seemed rather tired, and vomited large quantities of the same sour fluid as before; would not eat meat or drink milk, but drank water freely. Gave about ⅜ grain morphiæ sulphas hypodermically, and during the afternoon he seemed extremely sleepy and much disposed to lie down flat, but is nervous and easily alarmed by sudden noises, etc., etc. This condition lasted all day, and suppose it is the effect of the morphia.

November 21.—Died during night. Post-mortem showed a separation of the resection, which had evidently first torn out at the mesenteric attachment. Extensive peritonitis, intestines being agglutinated together and abdomen filled with fluid blood and fæces.

EXPERIMENT NO. 4.

November 23.—(Ball cal. 32.) Very large dog, weight about seventy-five pounds. Upon opening abdomen found a section of intestine for about six inches perforated in several places, the ball apparently having skipped along inside the gut; at another place the free edge was shot off and other portions of ileum grazed three times. The middle section was resected entirely, and closed perfectly; the shot edge was closed by trimming edge or side of the tube of intestine by a "V"-shaped cut towards the mesenteric attachment, and that also was closed fairly well. This was close to the left end of the pancreas. Each opening showed extrusion of contents. Many worms.

The operation lasted two hours, abdominal wound closed by two sets of sutures, one set through the muscular walls, the other bringing the skin in contact. Wound was covered with cotton, and bandage applied. Gave 20 gtt. deod. tinct. opium and a little water. Seemed to be doing well all P. M., and at night at six o'clock gave about 20 gtt. more of the opium, and left some water where he could get it.

November 24.—Dog still alive and very thirsty, but vomits the water soon after drinking; gave another dose of opium at night.

November 25.—Bandages have not been changed.

November 26.—Changed dressings; dog seems bright. Gave pint of milk; also opium, which he drank readily, but soon vomited. Gave more milk about noon, which he retained. In the afternoon he seemed much weaker. Gave deod. tincture of opium gtt. 20 at night.

Still lives, but is not strong. Lies in any position in which placed, and seems quite prostrated. Opium as before seems to revive him; he refuses milk, but drinks freely of water, which the stomach promptly rejects. In the afternoon, being no better, removed dressings, and although wound was quite healed made an opening for medium-sized drainage-tube, and let out about a quart of bloody serum; re-dressed the wound after injecting a weak carbolized solution of warm water into abdomen through tube. Gave a rectal injection of alcohol and water (1-2 1-2) warm and gtt. 20 of opium. Is getting very poor, but respiration is regular; pulse very weak and rapid.

November 28.—Gave enema of soapy water very weak. Washed out wound and inserted short drainage-tube and fresh bandages. About eleven o'clock had a passage from bowels of a large quantity of black, tarry, and badly smelling fæces, result of injection per rectum. At 2 P. M. was very weak. Gave enema of whisky and milk, warm, about 2 oz., and made a stew of small bits of beef and milk in whisky, which he ate greedily. Gave milk and whisky per rectum every three hours, also Valentine's extract of meat.

November 29.—Seems much stronger; had a semi-liquid passage from bowels. Gave enema every four hours of Valentine's extract, milk and whisky; and also fed pieces of raw meat in milk.

November 30.—Steady improvement; another passage which evidently came from above seat of operation. Fed him on raw steak, and gave whisky per rectum every four hours. Sutures through integument have ulcerated their way out; were removed and dog allowed to lick his wound, as he promptly tears off all bandages.

December 1.—Feeding as before, with steady improvement.

December 2.—Another passage from bowels during night Gave meat, about one-half pound every three hours, which he eats greedily; marked improvement daily in strength and appearance.

December 3.—Same improvement.

December 4.—Sent dog down stairs in basement.

December 10.—Alive and apparently in perfect health. External wound closed completely.

December 11.—Seems sick; refuses to eat; howls at night.

December 12.—Has marked symptoms of tetanus, and is in a state of rigidity with episthotonos.

December 13.—Died, and post-mortem showed an obstruction of intestine by a large mass of meat or a collection of various substances of a gritty consistency which completely obstructed and occluded bowels for some distance. The bowel was opened above this, and abdominal cavity filled with intestinal contents, and organs all adhered, as result of peritonitis; resection wound quite strongly united. Dog died from careless feeding and obstruction following adhesion of knuckle of intestine to omental stump.

### EXPERIMENT No. 5.

Monday, November 26.—(Ball cal. 32.) Medium-sized, well-conditioned and sturdy dog. Shot passed through a six-inch piece of intestine, making several perforations. Much fæcal matter free in abdomen, some opposite each opening. Many tapeworms.

November 27.—Made a resection of but one piece six inches in length, including both wounds. In this instance the larger silk sutures were used. After the continuity of the intestine was restored there was a great deal of bleeding from the interior of the abdomen, the origin of which could not find, but allowing the air to reach into all parts of the abdominal cavity, it ceased after considerable loss of blood. A portion of the omentum being filled with blood, it was ligated and removed. In closing abdominal wound was obliged to tear away a certain amount of fat which was closely adherent to interior wall of abdomen, along the line of the wound, in order to introduce sutures so they would not include the fatty mass. Introduced two drainage-tubes, and applied large pad of cotton. Gave some opium, about gtt. 15. P. M. dog seemed considerably shocked, but was quite thirsty. At night gave more opium.

November 17.—Cotton was soaked with fluid from drain-tubes. Removed dressings, and, upon getting him upon his feet, a small quantity of fluid escaped. In the evening washed out the abdomen with a warm solution of carbolic acid, about $\frac{1}{2}$, and applied dressing of gauze. During the day he vomited considerable milk and water which he had drank, and was evidently very weak. Gave about gtt. 20 of opium at night. Died at 8 A. M. on November 28. Post-mortem showed that sutures had parted at mesenteric edge, and death was from peritonitis. Mortification of edges of resection.

### EXPERIMENT NO. 6.

December 5.—(Ball cal. 32.) Died of hæmorrhage, after being shot, from wound of renal arteries, the ball perforating one kidney. Several perforations of the small intestines, all of them showing extrusion of contents. One large, round worm free in cavity.

### EXPERIMENT NO. 7.

December 7.—Died under ether.

### EXPERIMENT NO. 8.

December 7.—(Ball cal. No. 32). Ball opened one of the mesenteric arteries, and after resecting three pieces of intestine, and closing wound nicely (every perforation showed fæcal matter, worms, etc.); she died in less than nine hours from the shock. Great loss of blood; died of loss.

### EXPERIMENT NO. 9.

December 10.—(Ball cal. 32.) Gave morphine hypodermically at 9 A. M. Medium-sized female dog. Anæsthetized at 9 A. M., and shot at 9.30, first shot simply going through abdominal walls; second shot higher up and perforating spleen. Operation began at 10.15. Found abdomen full of blood, fæcal matter, and some worms. Removed spleen and large mass of omentum; ligated and removed one piece; resected about three inches in length; perforated in two places. Much hæmorrhage; operation concluded at twelve. Gave opium and whisky; much shocked. Died.

EXPERIMENT No. 10.

A well-nourished bull-dog (female), about twenty-five pounds in weight. Was anæsthetized about 9.15 A. M., and then shaved over the abdomen. Was shot at 9.45 by a 32-100 calibre revolver just posterior to the umbilicus, the bullet entering on the right side about three inches from the median line, the point of exit being in the corresponding situation on the opposite side. On opening abdomen found animal pregnant. There was one wound through the right cornu of the uterus, rupturing the membranes of one fœtal dog, and allowing the escape of the amniotic fluid into the peritoneal cavity. One of the smaller mesenteric arterial branches was cut, and the small intestine perforated in one place. The abdomen contained considerable blood on opening immediately after the shot, and there was slight extravasation of fæcal matter from the gut at openings. The vagina and uterine ligaments were ligated by single carbolized silk ligatures, and the large gravid uterus removed. The hæmorrhage in the mesentery having been checked, the wound in the intestine was resected, about two inches being removed. The free ends were united with the interrupted silk ligature. The peritoneal cavity was sponged out and washed with slightly carbolized warm water. The external wound was united with about ten silk ligatures, and dressed with iodoform and gauze, the whole being covered with oakum and bandaged. About half a grain of morphia was administered hypodermically; and at twelve the dog was allowed to come from the influence of ether. She showed marked symptoms of shock, but rallied in the afternoon. She died in the night. Post-mortem revealed hæmorrhage from the uterine stumps, and some peritonitis commencing.

### EXPERIMENT NO. 11.

A full-grown, healthy-appearing dog. Etherized at 9.30 A. M. Abdomen shaved and cleansed. Was shot at 10 A. M., still under the influence of ether, the bullet from a 32 S. & W. revolver passing transversely through the lower part of the abdomen. Was placed on table and kept partially anæsthetized until 10.45. The animal then presented signs of extreme loss of blood, feeble respiration and heart's action, cold extremities, pallid gums, etc. Abdomen was opened by large crucial incision and found to be filled with blood. Bleeding was ascertained to come from a divided mesenteric artery, and was readily checked by ligature. Clots were turned out, and two wounds of small intestine found. But slight extravasation of contents of bowel into the cavity, still some matter and worms found at openings. The intestine was resected at the site of each wound, about three inches being removed in each place. The cut ends of each were then united by about twelve interrupted silk sutures, so placed as to bring peritoneal surfaces in apposition. Intestines were then returned to their place, the cavity sponged out, and the external wound closed tightly with silk sutures. This was finished at twelve o'clock, the dog appearing moribund at its close, and remaining in a condition of collapse for about three hours. Reaction then took place, and he was able to stand and walk about. Second day, took some milk, which was vomited at once. This was repeated at intervals during second and third days. Dressings were changed on third day. No discharge from wound. On fourth day vomiting was increased, and was fæcal in character. Dog too weak to stand. Dressings changed again and wound found to be discharging purulent fluid. Died at 4 P. M. on fourth day. Post-mortem showed sero-purulent exudation in abdominal cavity, intestines glued together by adhesive lymph, wounds uniting well, and occlusion of the bowel in the neighborhood of one of them, from its having been sharply folded upon itself and bound in the position by the inflammatory exudate.

### EXPERIMENT NO. 12.

December 27, 1883.—The bullet, 32 cal., entered the abdomen on a line corresponding to the junction of the anterior and lateral surfaces of the abdomen, just in front of the hind leg, its point of exit on the other side being on the same line a little above the umbilicus.

On opening the abdomen it was found that the lower part of the jejunum was cut in two places within two inches of each other, and that there was considerable blood in the peritoneal cavity from these cut surfaces, there being no mesenteric vessels cut; also fæces and worms.

Both wounds were included in the parts excised, and the cut ends of the intestines were fastened together by three sutures, and then stitched to the abdominal parieties, thus forming an artificial anus.

Considerable shock was experienced, and owing to a desire to hasten the operation, the peritoneal cavity was not as carefully sponged as it should have been.

The dressing consisted of iodoform, protective and oakum. Of tinct. opii. deod. gtt. 20 were given by the mouth. The operation lasted two hours. On the following day he took a little nourishment; there was no tenderness, but some pus was squeezed from the point of exit of the bullet, the dog lying on that side.

Next day about one-half ounce of pus was forced from the point of exit of the bullet, the dog lying on that side, and by turning him on to the other side an equal amount was obtained from the point of entrance, but there was no suppuration from the wounds themselves.

He took a little nourishment and seemed to be in good condition, respiration being normal and pulse regular. He had a free urination from the bladder, and soft stools were passed from the artificial opening. He died during the night. Post-mortem revealed a large amount of septic material in the peritoneal cavity.

### EXPERIMENT No. 13.

Saturday, December 29.—(Ball calibre 32.) Medium-sized, middle-aged female dog. Gave with the anæsthetic about ⅜ grain of morphia hypodermically after shot. Abdomen found full of blood; seat of hæmorrhage found at one of the point ot perforation, of which there were two; from these issued fæcal matter, gas and worms; a medium-sized mesenteric artery having been shot off. All the intestines were drawn out of abdomen for examination, and it was found necessary to resect two portions which were a considerable distance apart, both places closing neatly and perfectly. Abdomen washed out and external wound closed by one set of sutures and a large pad of oakum laid over and held in place by roller first, and over all a many-tailed bandage. Gave 25 gtt. laudanum.

December 30.—Dog got loose during night and was running around very briskly; room very cold and disagreeable. (On the afternoon of the day of operation some person had opened the doors and windows and exposed the animal to a strong, cold draft for about two and one-half hours.) In the evening gave hypodermically morphine, when she vomited for first time and seemed very weak.

December 31.—Seemed lively and well all day; gave milk, which she would drink but could not retain. About noon gave an enema of Valentine's extract, and in the evening left a pan of milk.

January 1, 1884.—She seems as well as ever, but the floor of the room was profusely decorated with vomit. The milk was all gone. Gave an equivalent of an ounce of whisky, of alcohol and water per rectum, and left a supply of water, as she seemed very thirsty. Bandages changed for the first time since the operation. There had been but little discharge and the wound was in good condition. Applied a large pad of oakum and a wide roller as before.

January 2.—Seems quite exhausted. Gave alcohol and water (1-2 1-2) per rectum about four or five times a day in quantities of about 1 ounce; has a diarrhœa and vomits.

January 3.—Diarrhœa continues, but no vomiting. Has some appetite, and gave raw meat (steak) chopped fine, every two or three hours; also fresh milk, which she drinks readily.

January 4.—Seems quite well, and hungry; fed regularly and removed all dressings; wound in good condition. Removed all stitches and did not apply dressing again. Appetite good.

January 5.—Dog is seemingly well; has a voracious appetite. Much wasted in flesh, but appears strong.

January 6, 7, 8.—Fed her upon milk; also meat chopped fine and raw.

January 9.—She seemed well enough to be sent down cellar, where she continues gaining strength and flesh.

January 15.—Is perfectly well. Recovery.

EXPERIMENT No. 14.

January 9, 1884.—This dog was allowed some milk a short time previous to the operation, hence his stomach was distended.

The first bullet (32 calibre) grazed the abdomen walls, not entering the peritoneal cavity.

The second entered on a line corresponding to the junction of the anterior and lateral surfaces of the abdomen, a short distance in front of the hind leg, coming out a little nearer the median line, and two inches nearer the front leg.

On opening the abdomen it was found there was some hæmorrhage, mucus and particles of food in its cavity and on surface of stomach, and that the lower part of the stomach was wounded, the point of exit being two inches from the point of entrance, passing through the whole thickness of the stomach. There was no wound of the gut. The peritoneal surfaces were drawn together with cat-gut, by inverting the edges and using the continued suture.

Great care was taken in the *toilette du peritoine.* Immediately after closing the external wound he vomited half a pint of blood, mucus and milk. Time of operation was one hour and a half. Then he was given tincture of opii deod. gtts. xx.

The wound was dressed with iodoform, protective and oakum.

On the tenth was given nothing except a little water. On the eleventh he was given a little milk, which caused some disturbance. On the sixteenth the stitches were removed and no dressing applied, there being but slight discharge from the wound made by the incision and none from the bullet wounds. Recovered.

EXPERIMENT NO. 15.

Small dog, female, was anæsthetized and shot at 10:30 A. M. (S. & W. revolver, 32 calibre.) First wound passed through abdominal muscles only. Shot again immediately, bullet this time passing transversely through middle of abdomen. Opening made at once by linear incision. But little blood in cavity. All bleeding stopped upon exposure of intestines to air. Five wounds of small intestine found, all showing extravasation of contents. Two resections of five inches each were made to include all wounds. Cut ends were united by a continued catgut suture in each place. Intestines returned and abdominal incision united by silk sutures, after thoroughly washing out cavity by 2 per cent. solution of carbolic acid. The operation was finished at 10:30 A. M. Dog was laid in a warm place, apparently suffering but little from shock. External wound dressed with iodoform, covered by protective carbolized gauze, tow and a bandage. Animal died in about twenty hours. Was not given any food or medicine in that time. Post-mortem showed some small blood-clots about the wounds in the intestine. No serum or other fluids in cavity, and no signs of peritonitis. Death from shock.

EXPERIMENT NO. 16.

A dog of uncertain breed, about twenty pounds in weight, was shaved over the abdomen and anæsthetized at 10 A. M. Was shot in the abdomen in front of umbilicus, the bullet entering on the right side and coming out on the same side about two inches nearer the median line, not entering the abdominal cavity or wounding the peritonæum. Was shot again, the bullet entering on the right side, external and posterior to the first, and coming out on the opposite side, about two inches from median line. The calibre of the revolver was 32-100. Upon opening the abdominal cavity the peritonæum was found to be plowed across between the wounds of entrance and exit, and the spleen to be slightly nicked, the bullet having skirted the abdominal walls. The only hæmorrhage was from the external wounds and the spleen and track of bullet. The spleen was removed, its peritoneal connections being ligated by five silk ligatures. The small intestine was resected, about four inches being removed. The abdominal cavity was washed with warm carbolized water. The external wound was sewed up by about ten sutures. The dog came from under the influence of ether at 11:30 A. M. The wound was dressed externally with iodoform and oakum, and fifteen drops of deodorized tr. of opium administered by the mouth. A curious phenomenon was observed upon cutting out the spleen. The stomach and intestine became distended enormously with gas, extruding from the abdominal cavity and covering a large area of the operating-table. They were with difficulty returned with steady pressure. The dog died in the night from shock and hæmorrhages from splenic stumps.

### Experiment No. 17.

January 23.—(Ball cal. 32.) Good-sized coach dog. Bullet passed through abdominal walls without wounding intestines and just entering the peritoneal cavity, as was found after opening abdomen, the point of entrance and exit being on either side of the middle line and five inches apart. Removed the major portion of the greater omentum and also resected about six inches of the ileum and closed the wound by five sutures, the external wound being but two inches long.

January 24.—Seems inclined to be quiet all day; had defecated during the night and urinated very little; drinks but little water, and does not vomit it. Is by nature a very frisky dog, and do not think his extreme quiet very favorable.

January 25.—Seems quiet; no bloating of abdomen; removed bandages; re-applied dressings. Refused milk all day; also water.

January 26.—Gave small quantity of milk in the afternoon; re-applied the dressings which had been removed the day before; found the bullet wounds much puffed up, and that the stitches had slipped in two places, leaving a hole opening into abdomen large enough to admit little finger. The portion of intestine viewed through opening in external wound looked red and inflamed, but not badly so; little running from the wound. Filled it with iodoform and applied pad of oakum.

January 27.—Gave about one-half pound of meat and a quart of milk; seemed to be ready to get well.

January 28.—Fed meat and milk during day, and he seems to be rapidly getting well.

January 29.—Wound gaping, but discharged him to the cellar. Recovered.

EXPERIMENT NO. 18.

January 25, 1884.—This dog, a black and tan bitch, having been shaved the day before, was anæsthetized and shot.

The bullet, 32 calibre, passed directly through the abdomen about its middle, piercing the gravid uterus in two places, and cutting the gut longitudinally. No large vessels were cut. The uterine attachments were ligated *en masse* and the uterus removed. Contents of bowel found at site of wound in intestine.

During the time that an excision of the gut was being made, a profuse hæmorrhage occurred from the uterine stumps, before they could again be ligated by passing a suture through and ligating one-half at a time, the animal was almost exhausted from hæmorrhage.

The excision of the gut was then completed, and the cut ends stitched together with silk. The peritoneal cavity was then thoroughly washed out with slightly carbolized warm water, and the external wound closed. The dressing consisted of iodoform, gauze and oakum.

Of tinct. opii. deod., gtt. xx were given. Death occurred within ten hours after the operation, from effects of the hæmorrhage.

### EXPERIMENT NO. 19.

Dog was full-grown and apparently healthy. When the abdomen was exposed by shaving, two small abscesses, each the size of a filbert, superficially seated and non-inflammatory, were discovered. They were not disturbed. The animal was anæsthetized at 8:30 A. M:, and at once shot through the middle of abdomen with a 44 calibre revolver. The dog was placed upon the table, and a linear incision of about three inches made in the median line. It was there found that the ball had glanced upon the abdominal muscles, and instead of going through the mass of small intestines, had been deflected so that it just entered the peritoneal cavity beneath the linea alba, traversed the cavity for about an inch, producing a contused wound of a fold of intestines, and then entered the abdominal parietes to make its exit opposite the wound of entrance, about two inches from the linea alba. Only a small amount of blood was found in cavity. Although none of the intestines were wounded, a resection of about two inches from the middle of the ileum was made. The divided ends were united by about a dozen interrupted silk sutures. The cavity was washed thoroughly with a 1 per cent. sol. of carbolic acid, the intestines returned, and stitches were being placed in external wound, when the abdominal cavity was found to be filling with blood. Source of the hæmorrhage was found to be a branch of mesenteric artery at the site of the resection, which had commenced to bleed as soon as circulation was restored by warmth of abdomen. A ligature was applied, the intestine returned and the cavity again thoroughly washed out. The external wound was now closed by silk sutures, the wound dusted with iodoform, and dressed by applying a few thicknesses of carb. gauze, covering this with a mass of tow and a bandage over all. Animal appeared to suffer but little from shock. On morning of second day was given ¼ grain morphia with 1-100th grain atropia by the mouth.

On the third day appeared greatly prostrated, vomited at intervals, and a muco-purulent discharge was noticed coming from nostrils and eyes. Vomiting ceased on fourth day. Prostration and evidence of fever kept up to the morning of fifth day, when improvement began. Discharge from nostrils continued about ten days. On the fourth day a small quantity of milk was taken and retained. Loose discharge from bowels on fifth day slightly colored with blood. A rectal injection of alcohol; water was given on the sixth day. Dressings changed for the first time on sixth day. Wound appeared healthy and united in its deeper portions. Some pus from superficial part of wound from this time on, the dog ate milk regularly, and had regular and normal passages from bowels. On ninth day sutures were removed from external wound, which had entirely closed. On the thirteenth day, February 10, 1884, dog is apparently perfectly well; has been eating regularly of raw beef, and has begun to gain in flesh. On the evening of thirteenth day dog was well. Recovery.

EXPERIMENT No. 20.

February 2.—(Ball cal. 32.) Died from ether before any incision was made.

EXPERIMENT No. 21.

A strong black dog, about 20 lbs., was shaved over the abdomen and then etherized at 9.15 A. M. Was shot with a 38-100 calibre revolver through the abdomen about opposite the umbilicus, and five inches to the right of the median line, the point of exit being in a corresponding situation on the opposite side. Upon opening the abdominal cavity such a large amount of blood was found that it was necessary to enlarge the incision by a cross cut. A large mesenteric artery was found to be cut and was ligated. Another smaller one was treated in the same way. There were two wounds in the small intestine close together, about six inches intervening between them. Extravasation of contents from both. One was perforating and the other nicking the gut on the mesenteric side. Eight inches were removed, and the free extremities of the intestine united by interrupted silk sutures. There were three other wounds nicking the intestine which were sewed in the same manner without resection. The end of the cæcum, which is peculiarly shaped in dogs, was shot off. Stained mucus and some shreds at the opening. This was sewed, turning the cut end in. The spleen was cut in one place, which was left with one deeply-planted suture. A large fold of omentum was ligatured and removed. The abdomen was thoroughly washed with carbolized water, and the external wound united with about fifteen sutures. It was then then dressed with iodoform and oakum, ½ ounce of alcohol and 15 gtt. of deodorized tincture of opium were administered per rectum, and at 12.15 the dog was allowed to come from the influence of ether. The same amount of alcohol and opium were administered as before at 6 P. M. The dog died during the night. Post-mortem revealed no evidence of inflammation, and some slight bleeding from the spleen. The sutures in the intestine were in good condition. The piece of gut, about eight inches long, supplied by the mesenteric artery, which was cut by the bullet, was found to be completely mortified.

EXPERIMENT NO. 22.

February 12, 1884.—(Ball cal. 32.) Brindle bull dog. No attempt to sew up the holes in the intestines, of which there were about twenty. Died the day following. "Tilley's anæsthetizer." Every opening showed evidence of extrusion of contents.

EXPERIMENT NO. 23.

February 28, 1884.—Tilley's anæsthetizer. Died before operation from effects of ether.

EXPERIMENT NO. 24.

February 28, 1884.—(Ball cal. 44.) A short, strong Spitz dog. Bullet wounds of entrance and exit 4 in. apart. Intestine perforated in four places and abraded in one spot. Intestinal worms free in abdomen. Tape worms protruding from perforations.

Extravasation of contents of the bowel. No arteries divided by bullet. Resected one piece, (including three perforations) 12 in. length. Removed a V-shaped piece including the fourth perforation, and inverted the serous surfaces by interrupted sutures, the same as in complete section. The apex of the V (pointed to the attached border of the bowel) controlled the oozing from the abraded spot by small suture passed across mesenteric side of abrasion, the abrasion being the size of a copper cent, and on the side of intestine. Washed the intestines and abdomen cavity as clean as possible by stream of weak carbolized and pretty warm water from the irrigator; closed abdomen wound by five deeply-placed sutures about ⅓ inch apart; gave hypodermic injection of ¼ grain of morphia. Shock and little loss of blood. Omentum also removed.

February 29.—In morning seemed very lively and bright; gave some water, which was immediately rejected by stomach. During morning vomited foul-smelling fluid and two large chunks of meat. About 10 A. M. gave hypodermic injection of ½ grain morphia; in very few minutes he laid down and began to whine as though in pain, and threw up large quantity of offensively-smelling fluid. Died about 3 P. M.

Post-mortem.—Abdomen showed evidence of intestinal extravasation, all organs being bound together by peritoneal inflammation; extravasation of blood beneath peritoneal covering of intestines, and small clots adherent all along the length of ileum. The stumps of ligated mesentery and omentum were black. The seat of the operation showed adhesion of the serous surfaces, and water could be forced through the excised piece which was taken out by a cut six in. to each side of the stitches, without any leaking at seat of operation. The spot of abrasion was swollen and blue, and there had been a little hæmorrhage from it. The intestines generally were contracted, glued together, and pressed into prismoidal and other shapes. Stomach empty.

EXPERIMENT NO. 25.

February 28, 1884.—(Ball cal. 44.) "Tasso."

Bullet under skin opposite to point of entrance. Intestine riddled in about four places, for which a complete section 20 in. in length was removed and was nicely adjusted; another hole in the ascending colon was closed on each side by the continuous suture; the tip of the spleen being shot off, to arrest hæmorrhage a ligature was passed around proximal side of wound tight enough for that purpose, but yet not enough to cause death of the spleen tissue beyond ligature. The stumps of ligated mesentery being gathered upon a suture, were united to intestine near or about at the seat of the approximation of the divided ends; omentum removed; gave rectal injection of alcohol and water ½ ½ about. Each opening in bowel had more or less of the contents around it.

February 29.—Seemed very quiet all the morning, and was quite indisposed to move. Towards noon, gave him, about 11 o'clock, about 1 oz. of alcohol and water ½ ½ per rectum and some water to drink, which was at once vomited. Seemed very tired all day and disposed to lie stretched out before the heat of the register, and his breathing was entirely thoracic and by means of the cervical muscles. At 6 P. M. gave hypodermic injection of morph. gr. ⅜, and left water where he could drink.

March 1.—Seemed very weak all day; gave hypodermic injection of morph. ½ gr. twice, the last at night. About noon gave rectal injection of alcohol and water.

March 2.—Still alive, but very cold; listless and indifferent; gave morph. in A. M., and tied him up in blanket. Returned at nine P. M., and poor "Tasso" was in rigor mortis. I think the exposure to cold during the day (Sunday) which was a very wintry day, was in great measure the cause of his death. He refused to drink any milk during the day, and also seemed to have lost his thirst for water.

Post-mortem March 4.—Extensive peritonitis present; no separation of the united intestine to be found at the seat of operation.

EXPERIMENT No. 26.

March 3.—(44 cal. cartridge.) Large, fat and old bitch. Used Frank Gould's revolver, 44, and upon opening the abdomen, found four large rents in the intestines (every one of which showed extrusion of contents and some worms) at a considerable distance apart, and a very profuse hæmorrhage from the wound of exit, which was not discovered until the resections were made, of which two included the wounds in the gut, which was about shot off, and much bleeding took place before they were found and ligated. The animal was so fat and boggy that it was with the greatest difficulty that hæmorrhage could be controlled, and the beast was old and presented signs of cataract in both eyes. The bladder was greatly distended, and the structure of the intestines themselves seemed "sleazy," and the sutures tore out with readiness upon slight traction. Cleansed out abdomen as best we could by thorough washing, but a little bleeding was going on when the wound was closed in the abdomen, and the operation given up as a bad job of one-half hour's duration.

March 4.—Found dead.

EXPERIMENT NO. 27.

March 4.—(44 cartridge.) Medium-sized dog; died from shock on night of 4th.

EXPERIMENT NO. 28.

March 6.—(Ball cal. 22, revolver.) Very small, black and tan dog. Shot him with a 22 cartridge, and had to use three shots before could get a good perforation and but little bleeding. Resected a piece around the bullet-hole of ¾ inches long, cleaned abdomen and closed tightly; gave morph. gr. ¼.

March 7.—Seemed very bright.

March 8.—Gave little milk and morphia in evening.

March 10.—Milk.

March 11.—Sent down cellar to be fed on milk.

March 12, 13.—Very hearty, and eats ravenously of milk and very little meat.

March 14, 15.—Doing nicely. Recovery.

EXPERIMENT NO. 29.

March 6.—(Ball cal. 44.) Large, strong dog. Used 44 cartridge. Found the abdomen full of blood; spleen perforated, and intestines wounded in three or four places. From these issued fæces, gas, etc. Removed spleen, omentum, and resected about 12 in. gut, including all the holes but one, which was sewed up by continuous stitch. The animal having lost nearly all his blood by this time, and as death was sure to ensue, one of the mesenteric arteries was ligated to ascertain results.

March 7.—Found dead in morning.

Post-mortem.—Intestine black, but the animal had evidently not lived long enough to get any positive mortification of ligatured part, or any interesting appearance at all.

EXPERIMENT NO. 30.

March 10.—Tilley's Inhaler. Large chandler bitch. Killed by ether.

EXPERIMENT NO. 31.

March 10.—Tilley's Inhaler. Large Hastman dog. Killed by ether.

EXPERIMENT NO. 32.

March 10.—(Ball cal. 22, rifle.) Medium-sized bitch. Found three mesenteric arteries severed, and intestines riddled in many places and far apart. Perforations showed fæcal matter and tapeworms. Stopped bleeding and returned intestines without closing the perforations, and closed abdomen. No dressing but iodoform (C. T. P.) Spleen also removed, being perforated.

March 11.—Still alive, and sent down cellar.

March 13, A. M.—No better.

March 14, 15, A. M.—Vomiting, and refused food.

March 18.—Dead. Septic peritonitis. Pockets of fæces.

EXPERIMENT NO. 33.

March 10.—Small dog (yellow). Shot with 22 cal. rifle. Three perforations of small intestine, showing fæcal matter. Removed a 4-in. piece in two places, and brought ends together very closely; stitched mesenteric stumps to attached border of intestine. Removed omentum, tying tightly, and also putting in three side stitches connecting sides of stumps on each side of ligation with one another.

March 11.—Alive, but feverish and vomiting.

March 12.— Died. Separation at seat of operation, and stumps mortified. See specimen. Fæcal extravasation.

EXPERIMENT NO. 34.

March 12.—(Ball cal. 22 rifle.) Old, mangy bitch. Died under ether. Tilley's Inhaler. Perforation showed extravasation of contents, fæcal matter and worms.

EXPERIMENT NO. 35.

March 12.—Medium-sized, short-haired, yellow cur (white nose). Shot with 22-ball rifle. Upon opening the abdomen, found blood flowing from a rent in the side of spleen. This organ was three times the normal size, but no holes in the intestines anywhere, and no hole could be found in the abdominal wall on either side. The bullet lay next the abdominal muscles, and was cut out from the wound of entrance. Free bleeding from the laceration in the spleen, which was controlled by a continued suture. Resected six inches of intestine and closed abdomen. Also omentum removed. Iodoform and oakum dressing; gave morphia.

March 13.—¼ gr. morphia A. M.; very weak P. M.

March 14.—Morph. A. M.; very weak P. M. Temperature 102.

March 15.—Re-opened, but found intestine in solid mass and filled with badly-smelling fluid. Washed out as best I could and reclosed. Seat of operation showed mortification on one side, and stumps mesentery also were black and soft.

March 16.—Still alive. Morphia gr. ¼.

March 19.—Dead. Found intestinal worms in abdominal cavity.

EXPERIMENT NO. 36.

March 12.—Short, black, stumpy and very fat dog. Fired four 22-balls at the animal before was sure that any had entered.

Found abdomen full of blood; two perforations five inches apart, and two mesenteric arteries shot off near the gut. Each perforation showed extravasation, fæces and worms. Ligated the arteries; resected one piece, including both holes; sponged out abdominal cavity; removed omentum, also a large quantity of fat which hung to the inner wall of the belly, and closed wound. Iodoform and oakum dressing; gave morphia.

March 13.—Quite bright. Morphia A. M. and P. M.

March 14.—Dead. Post-mortem. Found considerable peritonitis and mortification of the ends of the intestines where they were stitched together. The stumps of mesentery and omentum also showed signs of mortification.

### EXPERIMENT NO. 37.

March 13.—Medium-sized brindle bitch. No. 22-ball rifle. Resected one piece six inches having two holes; removed omentum very little; hæmorrhage; gas and fæces from wounds; temperature 98 2-5 at close of operation.

March 14.—¼ gr. morphia A. M. Temperature 102 2-5. Morphia P. M.

March 15.—¼ gr. morphia P. M.; temperature 102 2-5.

March 16.—Morphia P. M.; temperature 102.

March 17.—Gave milk and morphia P. M.

March 19, 20.—Gave milk and morphia P. M.; seems well.

April 22.—Perfectly well; recovery.

### EXPERIMENT NO. 38.

March 13.—Medium-sized yellow dog (with bare spot on tail). No. 22-ball rifle; found one lateral hole which closed by continuous suture, and three holes which were included in one piece which was cut in half by mistake; two arteries gave some bleeding, but were ultimately controlled. Contents of bowel found at each wound. Abdomen closed while yet there was some oozing from the wound, which ceased when bandages were applied; omentum removed.

March 14.—Very weak and much prostrated. Refused to lie down, and can stand with difficulty on his feet. Gave morphia 3/8 hypodermically, morning and evening.

March 15.—Found dead. Post-mortem. Mortification at seat of operation, and escape of intestinal contents.

EXPERIMENT NO. 39.

March 14.—(Ball 22-cal. rifle.) Medium-sized brindle dog (wolf face); found three ragged holes. Resected one piece which included all openings, the piece being seven inches long; closed neatly, the omentum being removed also (many tapeworms and considerable fæcal matter from openings), then washed clean by irrigator. The entire ileum was inspected and sponged off, and returned to abdomen. Spleen also pulled out and inspected; gave morphia and dressings of iodoform and oakum.

March 15.—Some shock; morphia.

March 16.—Morphia P. M. only.

March 17.—Morphia P. M. only.

March 19.—A. M. morphia, stercoraceous vomiting, and seems very sick; P. M. is evidently dying.

March 20.—Vomiting has stopped, and seems much better. Died in the evening. P. M. Septic peritonitis. Post-peritoneal abscesses.

EXPERIMENT NO. 40.

March 14, 4.45 P. M.—A young, black, bitch pup; anæsthetized and abdomen opened without shooting; ligated a mesenteric artery and closed wound to be re-opened to-morrow afternoon and resect the part supplied by ligated vessel; gave morphia 3/8.

March 15.—Opened her at 3 P. M. in presence of Prof. Parkes.

Intestine supplied by the ligated artery seemed softer than normal and its mesentery showed inflammatory exudate considerable effusion; closed wound, gave morphia at night.

March 16.—Gave morphia, gr. ¼.

March 17.—Sent down stairs.

March 19.—Seems quite well.

April 22.—Perfectly well. Recovery.

EXPERIMENT NO. 41.

March 19.—(Ball 22-cal. rifle.) Medium-sized young bitch (black). Found one abrasion which closed up by continued suture and the intestine in another place was about shot off; fæcal matter scattered all about, worms divided; resected the piece about one inch long; irrigated the abdominal cavity; removed omentum and then found a hole or rather the tip of the spleen shot off which bled some and was controlled by two interrupted sutures; gave morphia, gr. ¼, and applied dressings.

March 18, 19.—Morphia, gr. ½.

March 20.—Seems bright, but very quiet.

April 22.—Quite well. Recovery.

EXPERIMENT NO. 42.

March 19.—Twenty-two ball rifle (brindle, white bitch). Found one abraded edge and one hole through free edge of ileum, from which contents issued. Closed one by continued suture and resected across the hole by cutting out a ¾ inch piece. Did not remove omentum. Gave morphia ⅜ and applied dressing.

March 18 and 19.—Morphia. Seems to have a paralysis of left fore-leg since operation and for two days past seems to be salivated.

March 20.—Better.

April 22.—Well. Recovery.

EXPERIMENT NO. 43.

March 20.—(Stub tail.) A small half-breed terrier dog. Twenty-two ball rifle. Found two perforations and one abrasion, extravasation of gas and stained mucus and shreds of matter from openings going through mucous coat. Made a resection including all three wounds, the excised piece being six inches long, closed very snugly and connected the ligated mesenteric stumps with the attached border of intestine by a single ligature passing through both stumps (but two sets of vessels having been ligated) and both sides of the mesenteric borders of the united ends of the gut; removed omentum and irrigated abdomen cavity freely with a 1 per cent. of carbolic acid solution. Dressings, oakum and iodoform; gave morphia, ¼. Recovery.

EXPERIMENT NO. 44.

March 20.—A young, shaggy, cur dog opened without shooting, and ligated three sets of branches from the superficial mesentery artery, close to the main artery, and also a good-sized anastomatic connection with an adjoining vasa intestini tenuis, which ran parallel with and along the attached border of the bowel. The intestines supplied by the vessels blanched immediately. Closed abdomen; gave morphia, gr. ¼; applied dressings. Recovered.

Died subsequently from ether during examination as to result of above operation six days after it. Found intestine perforated by stick of wood four inches long, rolled in twine. Had removed it, and was about to sew up wound when ether killed him.

EXPERIMENT NO. 45.

March 20.—A good-sized spaniel dog (old). Stopped breathing once from ether before shooting. Shot with a rifle twenty-two ball. Found the abdomen full of blood, two arteries having been shot off and the ileum perforated in four places; from each contents extruded, two being so near together and the wounds so great as to almost carry away an entire segment of the bowel, necessitating a removal of about twenty inches. Much bleeding, which took about half-an-hour to control, being from the ligated stumps and bullet-wounds; the tissues were very brittle, and so loaded with fat as to make the operation difficult. Died in one half-hour after closing abdomen wound.

---

PATHOLOGICAL SPECIMENS SHOWN.

1st. Section of ileum made 24 hours after operation; showing the sutures all covered with exudate. Union sufficiently firm to allow distention with water without leaking.

2nd. Sections of intestines made four, six and eight weeks after operation—the animal having fully recovered. The union is firm and solid throughout entire circumference of the bowel. No narrowing of tube, or disposition to formation of a stricture. Two of the specimens show several of the sutures ulcerating into the lumen of the bowel.

3rd. Several specimens showing mortification of distal ends of stumps, and also mortification of applied edges of the bowel from tight sutures and ligatures.

4th. Several specimens showing giving way of sutured bowel ends at the mesenteric junction, allowing extravasation causing fatal inflammation—sutures failed to include the muscular coat.

5th. Specimens showing many varieties of wounds produced by the bullet.

